# Quantum Confined High-Entropy Lanthanide Oxysulfide Colloidal Nanocrystals

**DOI:** 10.1021/acs.nanolett.2c01596

**Published:** 2022-10-04

**Authors:** Brendan Ward-O’Brien, Paul D. McNaughter, Rongsheng Cai, Amrita Chattopadhyay, Joseph M. Flitcroft, Charles T. Smith, David J. Binks, Jonathan M. Skelton, Sarah J. Haigh, David J. Lewis

**Affiliations:** † Department of Materials, University of Manchester, Oxford Road, Manchester M13 9PL, U.K.; ‡ Department of Chemistry, University of Manchester, Oxford Road, Manchester M13 9PL, U.K.; § Department of Physics and Astronomy and the Photon Science Institute, University of Manchester, Oxford Road, Manchester M13 9PL, U.K.

**Keywords:** high entropy, nanocrystal, quantum dot, lanthanide oxysulfides

## Abstract

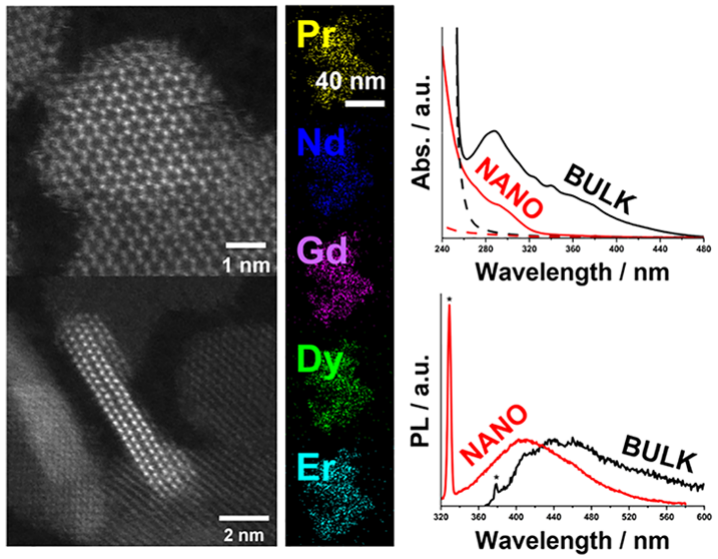

We have synthesized the first reported example of quantum confined high-entropy (HE) nanoparticles, using the lanthanide oxysulfide, Ln_2_SO_2_, system as the host phase for an equimolar mixture of Pr, Nd, Gd, Dy, and Er. A uniform HE phase was achieved *via* the simultaneous thermolysis of a mixture of lanthanide dithiocarbamate precursors in solution. This was confirmed by powder X-ray diffraction and high-resolution scanning transmission electron microscopy, with energy dispersive X-ray spectroscopic mapping confirming the uniform distribution of the lanthanides throughout the particles. The nanoparticle dispersion displayed a significant blue shift in the absorption and photoluminescence spectra relative to our previously reported bulk sample with the same composition, with an absorption edge at 330 nm and a λ_max_ at 410 nm compared to the absorption edge at 500 nm and a λ_max_ at 450 nm in the bulk, which is indicative of quantum confinement. We support this postulate with experimental and theoretical analysis of the bandgap energy as a function of strain and surface effects (ligand binding) as well as calculation of the exciton Bohr radiii of the end member compounds.

High-entropy (HE) compounds are a family of crystalline materials with high configurational entropy arising from at least five different elements in approximately equal atomic ratios randomly dispersed throughout a single crystalline phase.^1−3^ HE phases that have received much interest in recent years include oxides^4−6^ and alloys^1^ as well as chalcogenides,^7,8^ though this list continues to grow.^9−11^ The synthesis of HE materials with controllable composition at a variety of length scales to explore emergent properties associated with particle size is a particularly interesting challenge that is currently unmet.

Semiconducting inorganic materials that display emergent widening of the bandgap when the particle size falls below a critical size are known as quantum dots (QDs).^12,13^ The widening of the bandgap is a consequence of the physical confinement of excitons below their exciton Bohr radii in the bulk semiconductor. QDs have been applied to theranostics,^14^ LEDs,^15^ light harvesting,^16^ sensing,^17^ and catalysis.^18,19^ Quantum confined HE nanocrystals, *i.e.*, HE quantum dots, present a potentially vast range of accessible bandgaps through control of both nanocrystal composition and size, with potential applications as thermoelectrics and catalysts based on current studies on HE materials. HE materials have attracted significant attention as thermoelectric materials due to high levels of lattice distortion and consequent poor thermal conductivity.^20^ They have also been investigated for their promising catalytic properties due to the so-called “cocktail effect” caused by the simultaneous presence of multiple elements in close proximity to one another.^21^

The lanthanides, particularly the lanthanide oxysulfide system, Ln_2_SO_2_, represent a promising area of exploration for HE materials owing to the particular stability of the +3 oxidation state, as well as the relatively small variation in their crystallographic radii.^22^ They have found applications in a number of areas, such as multimodal medical imaging agents in the form of Gd_2_SO_2_ nanoparticles^23^ and as tunable bandgap materials in the form of bimetallic (Ce/Gd)_2_SO_2_ nanoparticles for solar cell applications.^24^ The 4*f*–4*f* transitions in the lanthanides means they also exhibit unique optical properties and so have found use as high-quantum-yield X-ray scintillators for exposure reduction.^25^

Bulk lanthanide oxysulfides have been produced by three routes, *viz*., by the partial sulfurization of the desired lanthanide oxide using a sulfur containing agent such as ammonium thiocyanate,^26^*via* the thermolysis of a molecular precursor such as a lanthanide dithiocarbamate derivative,^7,27^ and by calcination of urea-based precursors in the presence of elemental sulfur.^28,29^ Nanoparticles have also been produced, most commonly *via* solution-based synthetic procedures. During this process, inorganic lanthanide complexes are heated in a solvent or mixture of solvents that can act as capping agents for the resultant nanoparticles. As with bulk materials, this can be achieved with lanthanide salts and a sulfur source^24^ or with molecular precursors.^30^

The use of molecular precursors for the synthesis of nanomaterials has distinct advantages over other synthetic routes. The most significant are the prearranged atomic proximity of the elements present in the desired material and the ability to purify, characterize, or mix together in the desired ratios at the level of the molecule. Metal dithiocarbamate precursors, as used in this work, have been shown to allow the synthesis of a wide range of bulk materials, including main group, transition metal, and lanthanide sulfides, with compositions ranging from binary systems to high-entropy materials.^31−34^ However, there are limited examples of the production of nanoparticles using lanthanide dithiocarbamate precursors—to the best of our knowledge, the only prior published work explores the synthesis of Eu_2_SO_2_ and EuS nanoparticles.^30,35^

HE nanomaterials, specifically HE oxides, have been produced using techniques ranging from spray pyrolysis to mechanochemistry and sonochemistry.^4^ These methods require high processing temperatures due to the high stability of oxide materials and also require strategies to reduce residence times so as to avoid agglomeration. HE chalcogenide nanomaterials have been produced *via* a number of “top-down” synthetic techniques including ion exchange,^8^ extended elemental annealing,^36^ and high-temperature pulsed annealing.^37^ Unfortunately, both of these elemental annealing procedures require high temperatures, while the ion exchange route is highly specific to particular materials and has so far only been demonstrated using copper sulfide as a parent structure. A “bottom-up” synthesis approach for HE nanomaterials has not yet been reported and would be highly attractive in allowing new high-entropy materials to be built atom by atom. Such a bottom-up design would represent a true *tailored* synthetic approach, which is something that does not exist currently for HE nanomaterials and is limiting progress in the field.^38^

Focused on this goal, we report herein the first example of the synthesis of HE lanthanide oxysulfide nanoparticles with tunable composition and the potential for controlled morphology *via* a novel bottom-up strategy. The ability to control the composition of the nanoparticle *via* our precursor route allows for the direct comparison of a semiconducting HE material in bulk and nanoparticle form with near identical composition, thus allowing the first unambiguous observation of emergent properties produced purely by altering the size of the material.

In this study, we used the same selection of lanthanides as previously used in the synthesis of bulk HE Ln_2_SO_2_ materials.^7^ The chosen lanthanide elements are relatively closely grouped within the lanthanide series and have ionic radii within 0.1 Å.^22^ The theoretical maximum molar configurational entropy of mixing for an ideal HE Ln_2_SO_2_ sample, *i.e.*, with equimolar proportions of each constituent metal element and a perfectly random distribution of the lanthanide elements, is calculated to be *S*_m_ = 5.35 J K^–1^ mol^–1^ (see SI for details).^39^

In previous work by Stoll *et al.*, the thermogravimetric analysis profiles of the Ln precursors used in this work were discussed (see SI for detail on the synthesis and characterization of the molecular precursors). The Pr, Nd, and Gd precursors were shown to have similar decomposition profiles, decomposing fully between 250 and 400 °C.^27^ The Dy and Er precursors were found to follow a slightly different profile, decomposing at a lower temperature and doing so more gradually as the temperature increases, although the decomposition completes around 380 °C. We postulate that these different breakdown profiles may be responsible for the need to use a reduced amount of the Er precursor in order to produce nanoparticles with near equimolar elemental composition. As observed previously, even when using dried precursors, thoroughly degassed solvents and protective atmospheres, the oxophilicity of the lanthanides consistently leads to the production of the oxysulfide over the sulfide.^27^

Studying the decomposition of the solid precursors is informative but can only be used to guide the synthesis, as decomposition of the europium precursor is possible in solution at the significantly lower temperature of 290 °C as shown by Gao *et al.*(30) Relative to experiments done to produce bulk crystalline material, this is a substantial reduction in both the reaction time and reaction temperature.^27^ It was established that oleylamine, present both here and in the work of Gao *et al.*, plays a complex role in the nanoparticle synthesis and likely plays a part in the decomposition of the precursor.^40^

The X-ray diffraction pattern for the nanomaterial shows broad diffraction peaks, as expected for a nanocrystalline material, in positions that match our predicted pattern for the HE material (Figure 1). The peaks seen at 2θ ≈ 26, 29 and 47° are noticeably sharper than others, suggesting a significant proportion of plate-like nanoparticles extending outward in the (0001) plane. This is also expected, as oleic acid has been found to bind strongly to this plane and encourage lateral growth by stabilizing the (0001) surface.^41,42^ In order to deconvolute the effect of strain and crystallite size on the diffraction peak broadening, we carried out Williamson-Hall analysis on the XRD data.^43^ This suggests that the broadening is dominated by the small crystallite size rather than strain; the crystallite size is predicted to be 7.86 ± 0.89 nm, with strain of 0.55 ± 0.13%. Further discussion is provided in the SI.

**Figure 1:**
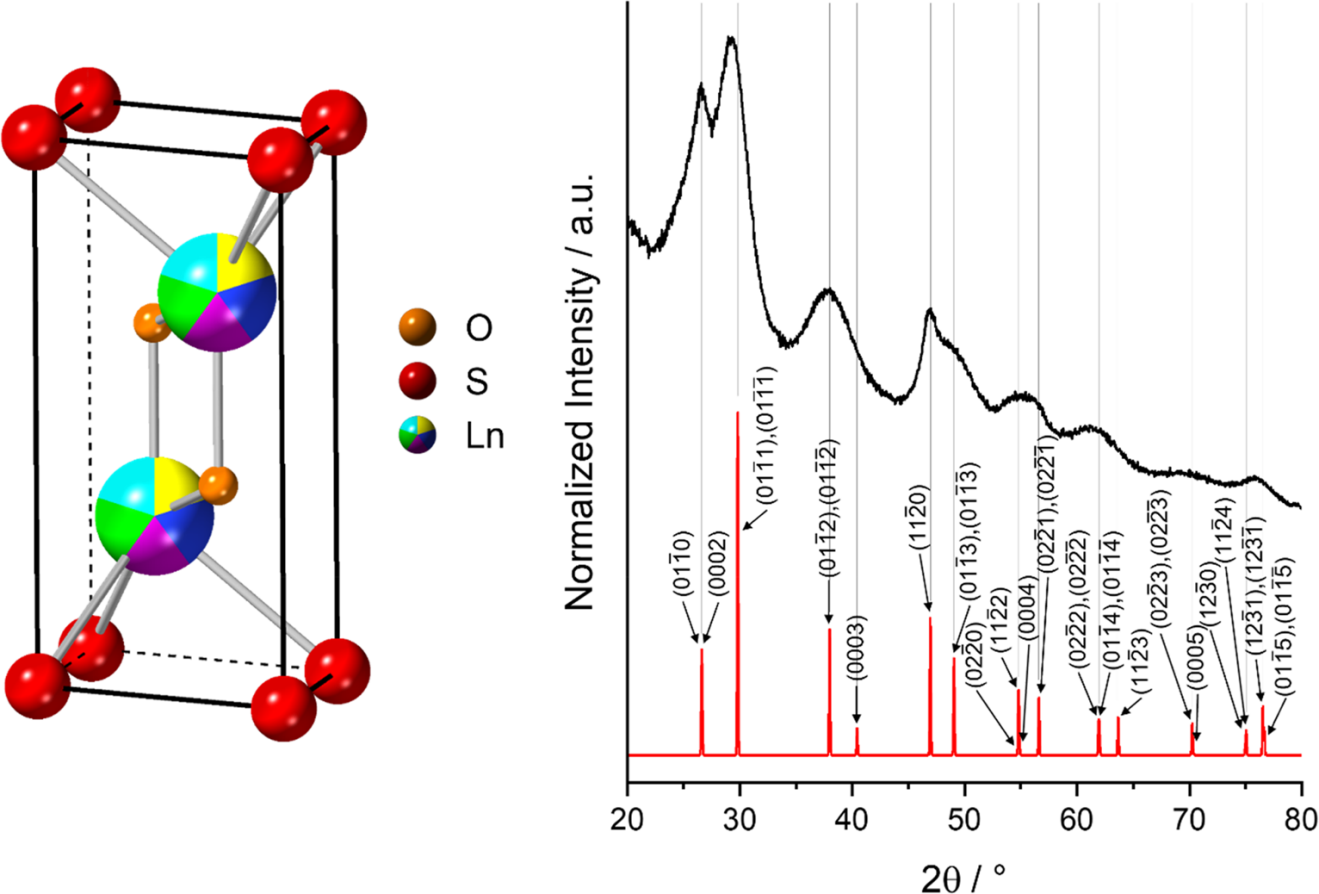
(Left) Predicted crystal structure of the HE Ln_2_SO_2_ material, where “Ln” represents the high-entropy site and has split occupancy between Pr, Nd, Gd, Dy and Er in equimolar ratios. The unit cell is based on the Gd_2_SO_2_ unit cell in the *P*3*m*1 space group, with altered occupancy of the Ln site and unit cell dimensions calculated by averaging over each of the constituent oxysulfides (*a* = 3.867 Å and *c* = 6.686 Å). The Gd_2_SO_2_ structure is taken from the International Centre for Diffraction Data (ICDD PDF card 00–026–1422). (Right) Powder XRD pattern of the nanoparticles (black) and the simulated diffraction pattern of the predicted Pr_0.4_Nd_0.4_Gd_0.4_Dy_0.4_Er_0.4_SO_2_ structure (red).

The high-resolution scanning transmission electron microscopy (HR-STEM) images confirm the formation of plate-like nanocrystals with the largest dimensions in the (0001) plane (Figure 2a–d). Analysis of the atomic resolution imaging data suggests good agreement with the predicted crystal structure as viewed along the [0001] direction (Figure 2d,g). The mean high-angle annular dark field (HAADF) STEM intensity of individual nanocrystals shows step changes, indicating that the nanoplates contain terraces of different thickness. Some orientations other than along the (0001) plane were also observed, but these appear to be partial rotations and so could not be indexed. The inset to Figure 2c shows the fast Fourier transform (FFT) image of the highlighted area, which matches predicted single crystal patterns along the [0001] zone-axis. Intensity profiles along the [1010] and [0110] directions are shown in the plot in Figure 2e, giving a direct measurement of the unit cell length averaged over the two directions as *a* = 3.72 ± 0.08 Å, which is contracted compared to our predicted *a* = 3.87 Å.

**Figure 2:**
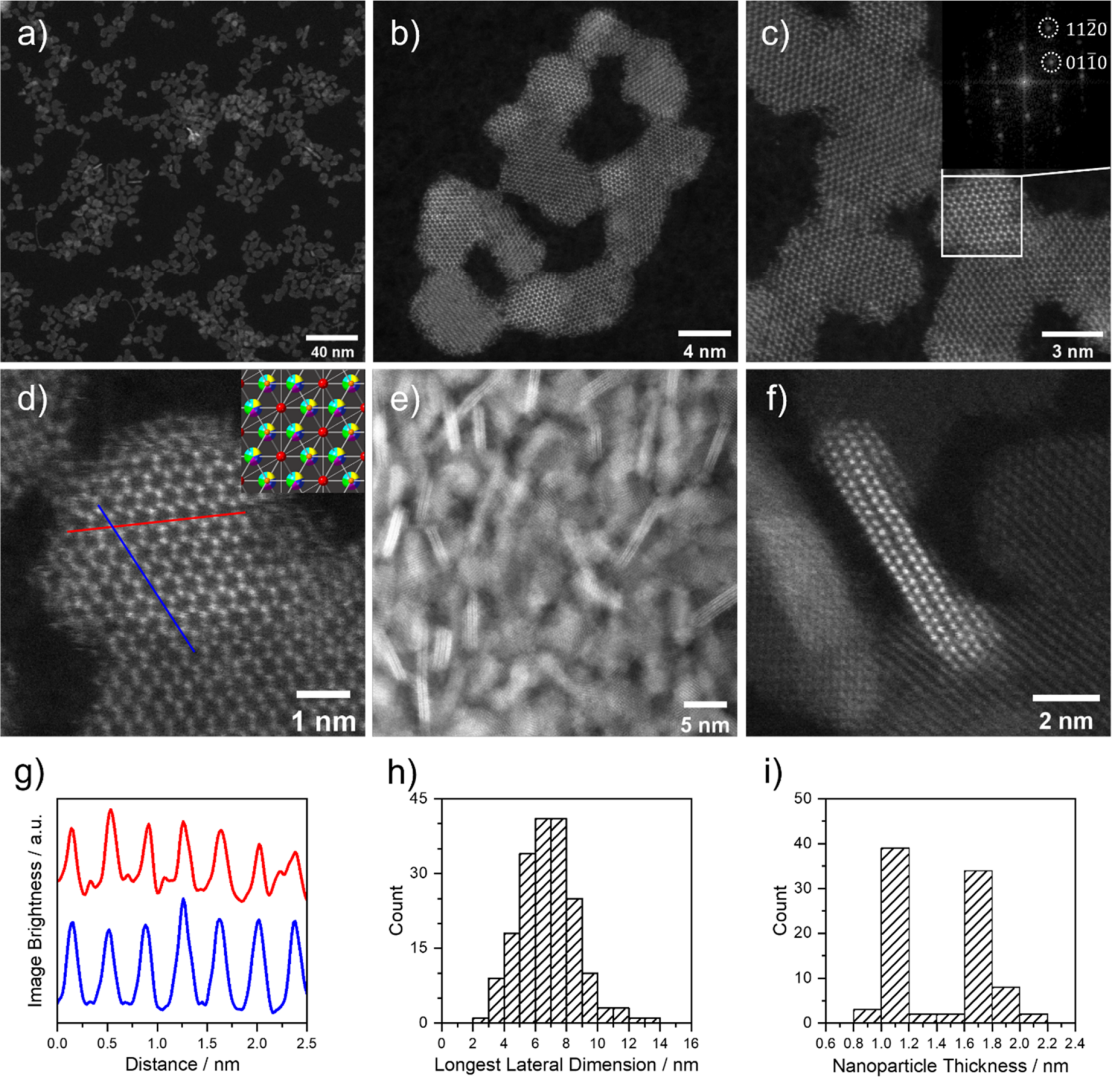
HAADF-STEM images of HE Ln_2_SO_2_ nanoparticles at lower (a,e) and higher magnifications (b–d,f). The inset to (c) shows the FFT image of the highlighted area, demonstrating that the particle is being viewed along the [0001] direction, and the inset to (d) shows the crystal structure viewed along the same direction as the STEM image. The plot in (g) shows the HAADF STEM intensity profiles along the [1010] and [0110] directions marked out in (d). The plots in (h) and (i) show respectively the particle size distribution of nanocrystals along the longest lateral dimension (*N* = 190) and the distribution of thicknesses (*N* = 90).

Figure 2f displays a size distribution of the mean lateral dimension of the (0001) basal plane determined from 190 particles in the image in Figure 2a, and Figure 2i also shows a distribution of nanoparticle thicknesses. From the latter analysis, we see a bimodal distribution of particle thicknesses, peaking between 1.0 and 1.2 and 1.6–1.8 nm, which corresponds to particles of two and three unit cells thick, respectively.

STEM energy dispersive X-ray (EDX) spectroscopic imaging confirmed a random distribution of all five lanthanide elements throughout the material with no signs of colocalization (Figure 3). Elemental quantifications from the summed spectrum, based on the Cliff-Lorimer method, allow the estimation of a chemical formula for the nanomaterial and hence quantification of an experimental entropy of mixing, *S*_m_. The chemical formula was determined to be Pr_0.42_Nd_0.43_Gd_0.42_Dy_0.41_Er_0.32_SO_2_, which equates to a *S*_m_ = 5.34 J K^–1^ mol^–1^, which is close to the theoretical maximum for this class of compound. S and O are not included in the compositional quantification due to the large error associated with EDX analysis of light elements, especially in the presence of the carbon support grid.

**Figure 3:**
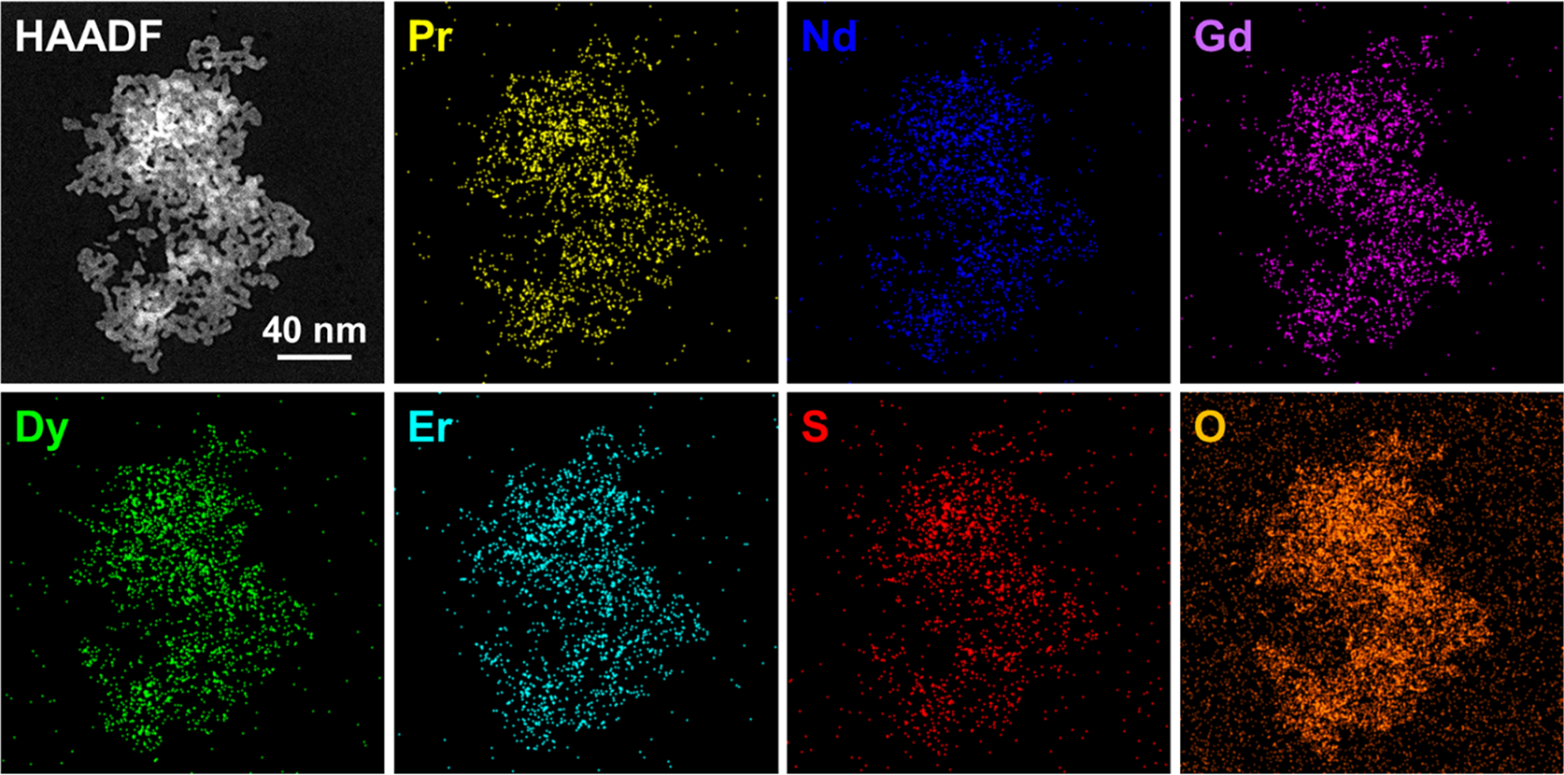
HAADF STEM image and STEM-EDX elemental maps showing the distribution of the different lanthanide elements throughout the nanoparticles. Spatial variations in X-ray intensity for all mapped elements are consistent and reflect the stacking of nanoparticles in some parts of the sample visible in the HAADF STEM image. The total electron beam fluence was 6.1 × 10^5^ e Å^–2^ when collecting the whole spectrum image. STEM-EDX elemental mapping at higher magnification was not possible due to limits on the maximum electron dose required to prevent electron beam damage as seen in Figure S3.

The optical absorption spectra of the nanoparticles display an absorption edge at approximately 330 nm (3.76 eV), with a significant increase in absorption intensity at approximately 250 nm (4.9 eV) as seen in Figure 4. A Tauc analysis was attempted, but a linear fit to obtain a bandgap was not possible, which we attribute to the bandgap being shifted into the UV-C region due to quantum confinement (*vide infra*).^44^ The absorption profile is significantly blue-shifted relative to that of the bulk samples produced in our previous work (Figure 4; *E*_g_ = 3.7 eV).^7^ This behavior is characteristic of the quantum confinement observed in quantum dots, where the increase in the bandgap is caused by the nanoparticle size being below the Bohr radius of the excitons in the bulk semiconductor.^7^

**Figure 4:**
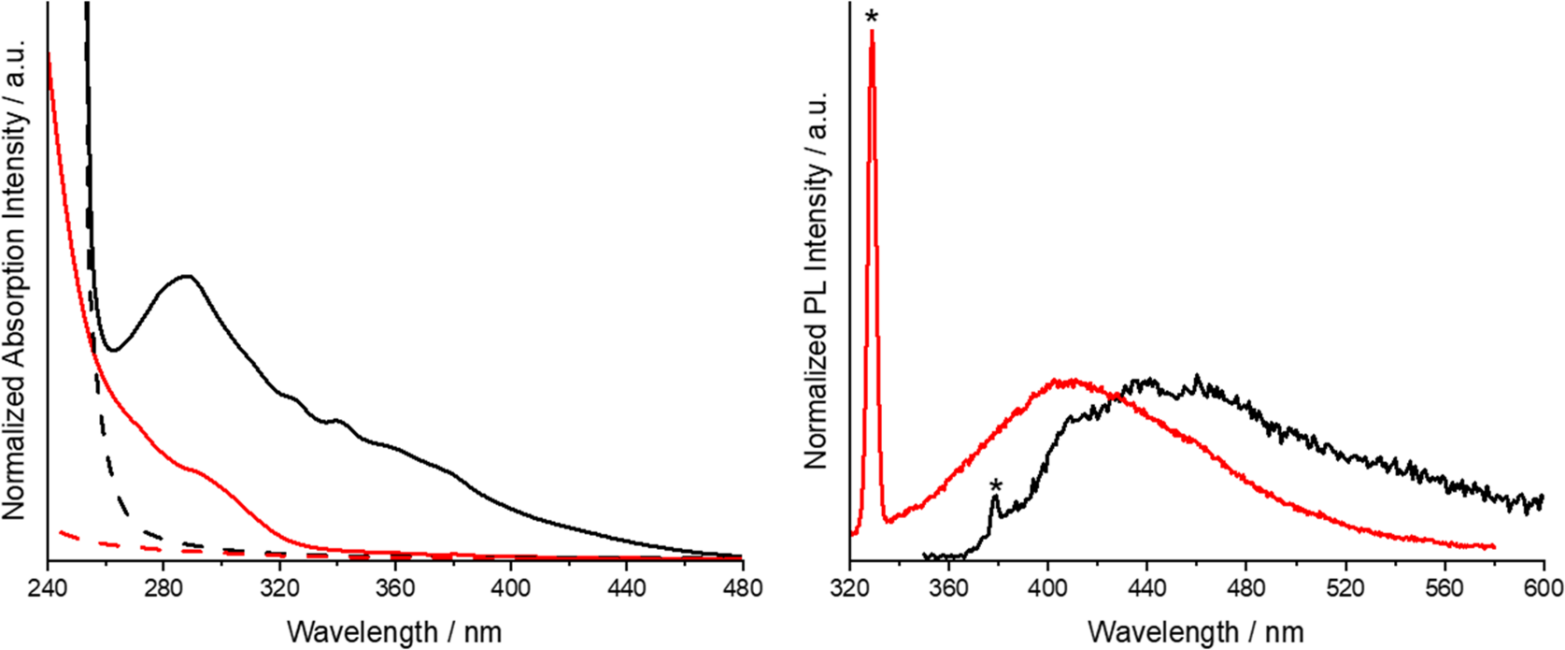
Comparison of the optical absorption and photoluminescence measured for the bulk HE phase in DMSO in our previous work (black) and for the HE nanoparticles in hexane in the present study (red). The dashed lines in the absorption spectra are the solvent blanks, and the Raman signals from the solvent in the PL spectra are highlighted with asterisks.^7^

The photoluminescence (PL) spectrum recorded using λ_ex_ = 300 nm gives a broad signal with a peak in intensity at approximately 410 nm. In comparison to reported work on bimetallic Gd_1.6_Ce_0.4_SO_2_ nanoparticles, the PL spectra of the HE nanoparticles are shifted to shorter wavelengths, and the features are significantly broader.^24^ The emission spectrum does, however, have the same general shape, with the intensity tailing off more gradually toward longer wavelengths with a shoulder-like feature at 450 nm. We tentatively attribute this broadening to the increased complexity of the band structure due to the large number of elements in the sample. We also analyzed the time-resolved photoluminescence of the nanocrystals and were able to fit the curve to three photoluminescence lifetimes, the longest of which was 4.1 ± 0.2 ns (see SI for fitted time-resolved PL decay curve), which suggests a number of pathways are available for the decay of the luminescent state.

Comparing the optical properties of the HE nanomaterials to those of the bulk Ln_2_SO_2_ material we reported previously reveals that both the absorption and PL spectra are significantly blue-shifted in the former (Figure 4).^7^ The absorption onset is substantially shifted to higher energy (ca. 1.3 eV) compared to the bulk data we previously published. To investigate whether this is a surface or strain effect, we performed theoretical modeling using density functional theory (DFT; see SI for further details). To quantify the impact of strain, we predicted the change of bandgap of the five single-component lanthanide oxysulfide endpoints over a range of compressions and expansions about the equilibrium volume covering strains of ±5%. This modeling, in combination with the strain of ε = 0.55% determined from Williamson-Hall analysis, suggests the strain in the nanoparticles would lead to a reduction of the bandgap on the order of 0.2 eV. The observed shift in the absorption onset is therefore substantially larger than would be expected from crystal strain alone. To investigate the effect of surface termination and confinement along the [0001] direction, corresponding to the major surface of the nanoparticles, we modeled a series of surface slabs of each of the five endpoints with 1–5 unit cells capped in the (0001) plane with either formate ([HCO_2_]^−^) or acetate ([CH_3_CO_2_]^−^). This choice is based on the previous finding that the introduction of oleic acid leads to the production of plates rather than rods, which implies that the (0001) plane is predominantly terminated by the organic acids, resulting in a lower surface free energy and faster growth perpendicular to this surface.^40,45^ These calculations found that slabs with two or more layers had a direct bandgap within 0.2 eV of the corresponding bulk phases, indicating that capping the surface with the organic acid and confining to a few layers along the [0001] direction do not significantly affect the bandgap and inferring strongly that the confinement dimension is the lateral size of the nanocrystals. While surface termination in the lateral dimension could also have an impact on the bandgap, given the higher bulk-to-surface ratio we would not expect this to be large. The calculated band structures and dielectric constants (relative permittivities) of the monometal endmembers were also used to estimate exciton Bohr radii (see SI). The radii were found to be on the order of a few nm (Table S1), demonstrating that the length scales of the high-entropy nanocrystals are indeed commensurate with the observation of quantum confinement.

In summary, our calculations show that neither strain effects nor surface effects have the required magnitude to account for the blue-shifts observed in the experimental optical spectra and that quantum confinement caused by the small lateral size of the nanocrystals (<10 nm) is likely to be responsible.

In this work, we have synthesized high-entropy lanthanide oxysulfide quantum dots *via* the thermolysis of a controlled mixture of molecular precursors in the presence of a mixture of capping agents. The nanoparticles obtained from this method were found to possess a very similar composition to the bulk lanthanide oxysulfides reported previously, allowing for direct comparison between a HE material with five metal components in the bulk and as a nanocrystal. The nanoparticles have significantly blue-shifted absorption and PL spectra, and from the consideration of composition, strain-related and surface effects on the bandgap this can be attributed to the decrease in particle size and, in turn, to quantum confinement. There is a great deal of potential for further work related to HE QD nanocrystals firstly in the wider sense of the appraisal of crystalline materials across the periodic table and their ability to form HE phases via our atom-by-atom assembly approach, and second in the control of morphology/particulate size anto tune the physical properties of these new systems. The most significant and deeper aspect resulting from this work therefore is the exciting prospect of novel classes of nanomaterial that may have emergent properties generated by high-entropy cocktail effects and quantum confinement effects acting in tandem. This will unlock new materials for a wide range of applications such as photonics and optoelectronics, photocatalysis and thermoelectric energy generation.

## Supporting Information Available

FTIR spectra of the molecular precursors and reagents, details of the calculation of the configurational entropy, details of the Williamson-Hall (W-H) analysis and the W-H plot, repeat high-resolution STEM-EDX and STEM-EDX measurements, details of the PL lifetime measurement and characterization, and details of the computational modeling and extended results (PDF)

## References

[ref1] Yeh J. W.; Chen S. K.; Lin S. J.; Gan J. Y.; Chin T. S.; Shun T. T.; Tsau C. H.; Chang S. Y. Nanostructured High-Entropy Alloys with Multiple Principal Elements: Novel Alloy Design Concepts and Outcomes. Adv. Eng. Mater. 2004, 6 (5), 299–303. 10.1002/adem.200300567.

[ref2] Yao Y.; Dong Q.; Brozena A.; Luo J.; Miao J.; Chi M.; Wang C.; Kevrekidis I. G.; Ren Z. J.; Greeley J.; Wang G.; Anapolsky A.; Hu L. High-entropy nanoparticles: Synthesis-structure-property relationships and data-driven discovery. Science 2022, 376 (6589), eabn3103 10.1126/science.abn3103. 35389801

[ref3] Buckingham M. A.; Ward-O’Brien B.; Xiao W.; Li Y.; Qu J.; Lewis D. J. High entropy metal chalcogenides: synthesis, properties, applications and future directions. Chem. Commun. 2022, 58 (58), 8025–8037. 10.1039/D2CC01796B. 35770747

[ref4] Sarkar A.; Breitung B.; Hahn H. High entropy oxides: The role of entropy, enthalpy and synergy. Scripta Mater. 2020, 187, 43–48. 10.1016/j.scriptamat.2020.05.019.

[ref5] Rost C. M.; Sachet E.; Borman T.; Moballegh A.; Dickey E. C.; Hou D.; Jones J. L.; Curtarolo S.; Maria J.-P. Entropy-stabilized oxides. Nat. Commun. 2015, 6 (1), 8485 10.1038/ncomms9485. 26415623 PMC4598836

[ref6] Tseng K.-P.; Yang Q.; McCormack S. J.; Kriven W. M. High-entropy, phase-constrained, lanthanide sesquioxide. J. Am. Ceram. Soc. 2020, 103 (1), 569–576. 10.1111/jace.16689.

[ref7] Ward-O’Brien B.; Pickering E. J.; Ahumada-Lazo R.; Smith C.; Zhong X. L.; Aboura Y.; Alam F.; Binks D. J.; Burnett T. L.; Lewis D. J. Synthesis of High Entropy Lanthanide Oxysulfides via the Thermolysis of a Molecular Precursor Cocktail. J. Am. Chem. Soc. 2021, 143 (51), 21560–21566. 10.1021/jacs.1c08995. 34923815

[ref8] McCormick C. R.; Schaak R. E. Simultaneous Multication Exchange Pathway to High-Entropy Metal Sulfide Nanoparticles. J. Am. Chem. Soc. 2021, 143 (2), 1017–1023. 10.1021/jacs.0c11384. 33405919

[ref9] Gild J.; Zhang Y.; Harrington T.; Jiang S.; Hu T.; Quinn M. C.; Mellor W. M.; Zhou N.; Vecchio K.; Luo J. High-Entropy Metal Diborides: A New Class of High-Entropy Materials and a New Type of Ultrahigh Temperature Ceramics. Sci. Rep. 2016, 6 (1), 37946 10.1038/srep37946. 27897255 PMC5126569

[ref10] Sarker P.; Harrington T.; Toher C.; Oses C.; Samiee M.; Maria J.-P.; Brenner D. W.; Vecchio K. S.; Curtarolo S. High-entropy high-hardness metal carbides discovered by entropy descriptors. Nat. Commun. 2018, 9 (1), 4980 10.1038/s41467-018-07160-7. 30478375 PMC6255778

[ref11] Nemani S. K.; Zhang B.; Wyatt B. C.; Hood Z. D.; Manna S.; Khaledialidusti R.; Hong W.; Sternberg M. G.; Sankaranarayanan S. K. R. S.; Anasori B. High-Entropy 2D Carbide MXenes: TiVNbMoC3 and TiVCrMoC3. ACS Nano 2021, 15 (8), 12815–12825. 10.1021/acsnano.1c02775. 34128649

[ref12] Ledoux G.; Gong J.; Huisken F.; Guillois O.; Reynaud C. Photoluminescence of size-separated silicon nanocrystals: Confirmation of quantum confinement. Appl. Phys. Lett. 2002, 80 (25), 4834–4836. 10.1063/1.1485302.

[ref13] García de Arquer F. P.; Talapin D. V.; Klimov V. I.; Arakawa Y.; Bayer M.; Sargent E. H. Semiconductor quantum dots: Technological progress and future challenges. Science 2021, 373 (6555), eaaz8541 10.1126/science.aaz8541. 34353926

[ref14] Lv G.; Guo W.; Zhang W.; Zhang T.; Li S.; Chen S.; Eltahan A. S.; Wang D.; Wang Y.; Zhang J.; Wang P. C.; Chang J.; Liang X.-J. Near-Infrared Emission CuInS/ZnS Quantum Dots: All-in-One Theranostic Nanomedicines with Intrinsic Fluorescence/Photoacoustic Imaging for Tumor Phototherapy. ACS Nano 2016, 10 (10), 9637–9645. 10.1021/acsnano.6b05419. 27623101 PMC5359086

[ref15] Shu Y.; Lin X.; Qin H.; Hu Z.; Jin Y.; Peng X. Quantum Dots for Display Applications. Angew. Chem., Int. Ed. 2020, 59 (50), 22312–22323. 10.1002/anie.202004857. 32421230

[ref16] Duan L.; Hu L.; Guan X.; Lin C.-H.; Chu D.; Huang S.; Liu X.; Yuan J.; Wu T. Quantum Dots for Photovoltaics: A Tale of Two Materials. Adv. Energy Mater. 2021, 11 (20), 2100354 10.1002/aenm.202100354.

[ref17] Chern M.; Kays J. C.; Bhuckory S.; Dennis A. M. Sensing with photoluminescent semiconductor quantum dots. Methods Appl. Fluoresc. 2019, 7 (1), 012005 10.1088/2050-6120/aaf6f8. 30530939 PMC7233465

[ref18] Bear J. C.; Hollingsworth N.; McNaughter P. D.; Mayes A. G.; Ward M. B.; Nann T.; Hogarth G.; Parkin I. P. Copper-Doped CdSe/ZnS Quantum Dots: Controllable Photoactivated Copper(I) Cation Storage and Release Vectors for Catalysis. Angew. Chem., Int. Ed. 2014, 53 (6), 1598–1601. 10.1002/anie.201308778. PMC4138996 24376131

[ref19] Wakerley D. W.; Kuehnel M. F.; Orchard K. L.; Ly K. H.; Rosser T. E.; Reisner E. Solar-driven reforming of lignocellulose to H2 with a CdS/CdOx photocatalyst. Nat. Energy 2017, 2 (4), 17021 10.1038/nenergy.2017.21.

[ref20] Jiang B.; Yu Y.; Cui J.; Liu X.; Xie L.; Liao J.; Zhang Q.; Huang Y.; Ning S.; Jia B.; Zhu B.; Bai S.; Chen L.; Pennycook S. J.; He J. High-entropy-stabilized chalcogenides with high thermoelectric performance. Science 2021, 371 (6531), 830 10.1126/science.abe1292. 33602853

[ref21] Nguyen T. X.; Su Y.-H.; Lin C.-C.; Ting J.-M. Self-Reconstruction of Sulfate-Containing High Entropy Sulfide for Exceptionally High-Performance Oxygen Evolution Reaction Electrocatalyst. Adv. Funct. Mater. 2021, 31 (48), 2106229 10.1002/adfm.202106229.

[ref22] Shannon R. D. Revised effective ionic radii and systematic studies of interatomic distances in halides and chalcogenides. Acta Crystallogr., Sect. A 1976, 32 (5), 751–767. 10.1107/S0567739476001551.

[ref23] Osseni S. A.; Lechevallier S.; Verelst M.; Perriat P.; Dexpert-Ghys J.; Neumeyer D.; Garcia R.; Mayer F.; Djanashvili K.; Peters J. A.; Magdeleine E.; Gros-Dagnac H.; Celsis P.; Mauricot R. Gadolinium oxysulfide nanoparticles as multimodal imaging agents for T2-weighted MR, X-ray tomography and photoluminescence. Nanoscale 2014, 6 (1), 555–564. 10.1039/C3NR03982J. 24241248

[ref24] Larquet C.; Nguyen A.-M.; Glais E.; Paulatto L.; Sassoye C.; Selmane M.; Lecante P.; Maheu C.; Geantet C.; Cardenas L.; Chanéac C.; Gauzzi A.; Sanchez C.; Carenco S. Band Gap Engineering from Cation Balance: The Case of Lanthanide Oxysulfide Nanoparticles. Chem. Mater. 2019, 31 (14), 5014–5023. 10.1021/acs.chemmater.9b00450.

[ref25] Buchanan R. A.; Finkelstein S. I.; Wickersheim K. A. X-Ray Exposure Reduction Using Rare-Earth Oxysulfide Intensifying Screens. Radiology 1972, 105 (1), 185–190. 10.1148/105.1.185. 5057301

[ref26] Sotnikov A. V.; Bakovets V. V.; Sokolov V. V.; Filatova I. Y. Lanthanum oxide sulfurization in ammonium rhodanide vapor. Inorg. Mater. 2014, 50 (10), 1024–1029. 10.1134/S0020168514100173.

[ref27] Boncher W. L.; Regulacio M. D.; Stoll S. L. Thermolysis of lanthanide dithiocarbamate complexes. J. Solid State Chem. 2010, 183 (1), 52–56. 10.1016/j.jssc.2009.10.003.

[ref28] Bagheri A.; Rezaee Ebrahim Saraee K.; Shakur H. R.; Zamani Zeinali H. Synthesis and characterization of physical properties of Gd2O2S:Pr3+ semi-nanoflower phosphor. Appl. Phys. A: Mater. Sci. Process. 2016, 122 (5), 553 10.1007/s00339-016-0058-z.

[ref29] Sun W.; Yang X.; Yu M.; Wang L.; Zhang Q. Experimental and theoretical studies on the stable synthesis of a laser protective coating material erbium oxysulfide. J. Mater. Sci. Mater. Electron. 2018, 29 (3), 2406–2415. 10.1007/s10854-017-8159-9.

[ref30] Zhao F.; Yuan M.; Zhang W.; Gao S. Monodisperse Lanthanide Oxysulfide Nanocrystals. J. Am. Chem. Soc. 2006, 128 (36), 11758–11759. 10.1021/ja0638410. 16953606

[ref31] Murtaza G.; Alderhami S.; Alharbi Y. T.; Zulfiqar U.; Hossin M.; Alanazi A. M.; Almanqur L.; Onche E. U.; Venkateswaran S. P.; Lewis D. J. Scalable and Universal Route for the Deposition of Binary, Ternary, and Quaternary Metal Sulfide Materials from Molecular Precursors. ACS Appl. Energy Mater. 2020, 3 (2), 1952–1961. 10.1021/acsaem.9b02359. 32296758 PMC7147260

[ref32] Alanazi A. M.; Alam F.; Salhi A.; Missous M.; Thomas A. G.; O’Brien P.; Lewis D. J. A molecular precursor route to quaternary chalcogenide CFTS (Cu2FeSnS4) powders as potential solar absorber materials. RSC Adv. 2019, 9 (42), 24146–24153. 10.1039/C9RA02926E. 35527861 PMC9069629

[ref33] Sarker J. C.; Hogarth G. Dithiocarbamate Complexes as Single Source Precursors to Nanoscale Binary, Ternary and Quaternary Metal Sulfides. Chem. Rev. 2021, 121 (10), 6057–6123. 10.1021/acs.chemrev.0c01183. 33847480

[ref34] Malik M. A.; Afzaal M.; O’Brien P. Precursor Chemistry for Main Group Elements in Semiconducting Materials. Chem. Rev. 2010, 110 (7), 4417–4446. 10.1021/cr900406f. 20481563

[ref35] Zhao F.; Sun H.-L.; Su G.; Gao S. Synthesis and Size-Dependent Magnetic Properties of Monodisperse EuS Nanocrystals. Small 2006, 2 (2), 244–248. 10.1002/smll.200500294. 17193029

[ref36] Cavin J.; Ahmadiparidari A.; Majidi L.; Thind A. S.; Misal S. N.; Prajapati A.; Hemmat Z.; Rastegar S.; Beukelman A.; Singh M. R.; Unocic K. A.; Salehi-Khojin A.; Mishra R. 2D High-Entropy Transition Metal Dichalcogenides for Carbon Dioxide Electrocatalysis. Adv. Mater. 2021, 33 (31), 2100347 10.1002/adma.202100347. 34173281

[ref37] Cui M.; Yang C.; Li B.; Dong Q.; Wu M.; Hwang S.; Xie H.; Wang X.; Wang G.; Hu L. High-Entropy Metal Sulfide Nanoparticles Promise High-Performance Oxygen Evolution Reaction. Adv. Energy Mater. 2021, 11 (3), 2002887 10.1002/aenm.202002887.

[ref38] Koo W.-T.; Millstone J. E.; Weiss P. S.; Kim I.-D. The Design and Science of Polyelemental Nanoparticles. ACS Nano 2020, 14 (6), 6407–6413. 10.1021/acsnano.0c03993. 32469489

[ref39] Hillert M. Phase equilibria, phase diagrams and phase transformations: their thermodynamic basis, 2nd ed.; Cambridge University Press: Cambridge, 2008.

[ref40] Mourdikoudis S.; Liz-Marzán L. M. Oleylamine in Nanoparticle Synthesis. Chem. Mater. 2013, 25 (9), 1465–1476. 10.1021/cm4000476.

[ref41] Zhao F.; Gao S. Pyrolysis of single molecular precursor for monodisperse lanthanide sulfide/oxysulfide nanocrystals. J. Mater. Chem. 2008, 18 (9), 949–953. 10.1039/B713636F.

[ref42] Asuigui D. R.; Atif R.; Swanson J.; Glaser P.; Garskaite E.; Žarkov A.; Stoll S. L. Chapter 7 - Synthesis of lanthanide chalcogenide nanoparticles. Nanomaterials via Single-Source Precursors 2022, 219–243. 10.1016/B978-0-12-820340-8.00012-5.

[ref43] Williamson G. K.; Hall W. H. X-ray line broadening from filed aluminium and wolfram. Acta Metall. 1953, 1 (1), 22–31. 10.1016/0001-6160(53)90006-6.

[ref44] Tauc J.; Grigorovici R.; Vancu A. Optical Properties and Electronic Structure of Amorphous Germanium. Phys. Status Solidi (b) 1966, 15 (2), 627–637. 10.1002/pssb.19660150224.

[ref45] De Yoreo J. J.; Vekilov P. G., 3. Principles of Crystal Nucleation and Growth. In Biomineralization; Dove P. M.; De Yoreo J. J.; Weiner S., Eds.; De Gruyter: Berlin, 2018; pp 57–94.

