# Supplementary material for: Quantum Confined High-Entropy Lanthanide Oxysulfide Colloidal Nanocrystals
Source: Nano Lett. 2022 Oct 4;22(20):8045–51. doi: 10.1021/acs.nanolett.2c01596 (PMC9614967; doi:10.1021/acs.nanolett.2c01596)
Supplement: Supplementary file 1 — nl2c01596_si_001.pdf [file nl2c01596_si_001.pdf]

SUPPORTING INFORMATION

Quantum Confined High Entropy Lanthanide Oxysulfide Colloidal Nanocrystals

Brendan Ward-O'Brien,[1†] Paul D. McNaughter,[2†] Rongsheng Cai,[1] Amrita Chattopadhyay,[2] Joseph M. Flitcroft,[2] Charles T. Smith,[3] David J. Binks,[3] Jonathan M. Skelton,[2] Sarah J. Haigh,[1] David J. Lewis[1]*

[1]Department of Materials, University of Manchester, Oxford Road, M13 9PL, UK

[2]Department of Chemistry, University of Manchester, Oxford Road, M13 9PL, UK

[3]Department of Physics and Astronomy and the Photon Science Institute, University of Manchester, Oxford Road, M13 9PL, UK

†Authors contributed equally

E-mail: david.lewis-4@manchester.ac.uk

EXPERIMENTAL

Chemicals. All chemicals were purchased from Sigma-Aldrich. 1,10-phenanthroline (phen, ≥ 99.0%), sodium diethyldithiocarbamate trihydrate ($NaS_2CNEt_2 \cdot 3H_2O$, ≥ 99.0%), praseodymium(III) nitrate hexahydrate ($Pr(NO_3)_3 \cdot 6H_2O$, 99.9%), neodymium(III) nitrate hexahydrate ($Nd(NO_3)_3 \cdot 6H_2O$, 99.9%), gadolinium(III) nitrate hexahydrate ($Gd(NO_3)_3 \cdot 6H_2O$, 99.9%), dysprosium(III) nitrate hydrate ($Dy(NO_3)_3 \cdot xH_2O$, 99.9%), erbium(III) nitrate pentahydrate ($Er(NO_3)_3 \cdot 5H_2O$, 99.9%), oleylamine ($C_8H_{17}CH{=}CH(CH_2)_7CH_2NH_2$, technical grade, 70%), oleic acid ($C_8H_{17}CH{=}CH(CH_2)_7CO_2H$, technical grade, 90%), octadecene ($C_{16}H_{33}CH{=}CH_2$, technical grade, 90%), methanol (99.9%), acetonitrile (99.9%), hexane (≥ 97.0%) and acetone (≥ 95%) were used as purchased without further purification.

Precursor Synthesis. The [$Ln(S_2CNEt_2)_3$.phen] precursor was prepared as previously reported.(ref 7 in main manuscript). Briefly, sodium diethyldithiocarbamate (3 mmol, 0.68 g) and 1,10-phenanthroline (1 mmol, 0.18 g) were dissolved in 35 mL of a 5:2 *v/v* mixture of MeOH:MeCN with gentle heating to aid dissolution. 1 mmol of the desired lanthanide nitrate, where Ln = Pr, Nd, Gd, Dy, Er, was dissolved in 10 mL of MeOH and added dropwise to the first solution. The reaction mixture was cooled to 4 °C for an hour, resulting in a precipitate that was collected by vacuum filtration and washed with cold MeCN before being dried *in vacuo*.

Characterization of precursors.

[$Pr(S_2CNEt_2)_3$.(phen)]; Fourier transform infrared (FTIR) (solid) ν_{max}/cm^{-1}: 3044 (w), 2972 (w), 2928 (w), 2902 (w), 2869 (w), 1622 (w), 1588 (w). Elemental analysis (EA) measured % (expected %): C, 42.1 (42.3); H, 5.0 (5.0); N, 9.0 (9.1); S, 24.8 (25.1).

[$Nd(S_2CNEt_2)_3$.phen)]; FTIR (solid) ν_{max}/cm^{-1}: 3044 (w), 2973 (w), 2928 (w), 2903 (w), 2869 (w), 1623 (w), 1589 (w). EA measured % (expected %): C, 41.5 (42.2); H, 4.9 (5.0); N, 8.9 (9.1); S, 24.5 (25.0).

[$Gd(S_2CNEt_2)_3$.(phen)]; FTIR (solid) ν_{max}/cm^{-1}: 3044 (w), 2973 (w), 2928 (w), 2903 (w), 2869 (w), 1624 (w), 1589 (w). EA measured % (expected %): C, 40.48 (41.5); H, 4.8 (4.9); N, 8.7 (9.0); S, 23.9 (24.6).

[$Dy(S_2CNEt_2)_3$.(phen)]; FT-IR (solid) ν_{max}/cm^{-1}: 3045 (w), 2974 (w), 2928 (w), 2903 (w), 2869 (w), 1624 (w), 1589 (w). EA measured % (expected %): C, 39.75 (41.2); H, 4.7 (4.9); N, 8.5 (8.9); S, 23.22 (24.4).

[$Er(S_2CNEt_2)_3$.(phen)]; FT-IR (solid) ν_{max}/cm^{-1}: 3045 (w), 2975 (w), 2928 (w), 2903 (w), 2869 (w), 1625 (w), 1590 (w). EA measured % (expected %): C, 37.3 (40.9); H, 4.8 (4.8); N, 8.2 (8.8); S, 22.00 (24.3).

Nanoparticle Synthesis. The nanoparticles were prepared *via* the solution based thermolysis reported by Zhao *et al.*(ref 30 in main manuscript) Briefly, oleylamine (23.9 mmol, 6.4 g), oleic acid (9.6 mmol, 2.7 g) and octadecene (9.5 mmol, 2.4 g) were mixed with a 1:1:1:1:0.66 molar ratio of the [$Ln(S_2CNEt_2)_3$.phen] species (73 mg, 74 mg, 75 mg, 76 mg and 51 mg of the Pr, Nd, Gd, Dy and Er precursors respectively). The mixture was placed under vacuum at room temperature for 5 minutes before replacing the atmosphere with nitrogen. This process was repeated three times. The mixture was then heated to 70 °C and held under vacuum for 15 minutes, during which the [$Ln(S_2CNEt_2)_3$.phen] complexes dissolved to form a pale yellow solution. The reaction mixture was then heated to 290 °C over the course of 30 minutes and

held at this temperature for 1 hour. At approximately 140 °C the solution changed from pale yellow to dark green, then returned to yellow during heating. The solution was then cooled to room temperature rapidly to halt nanoparticle growth.

The reaction mixture was found to be robust to being handled in air to isolate the nanoparticles from the reaction mixture. An excess of acetone was added to the reaction mixture resulting in a colourless precipitate that was collected by centrifugation at 8000 × *g* for 5 minutes and re-suspended in approximately 4 mL of hexane. The process of precipitation, removal of the supernatant and re-suspension in hexane was repeated two more times to ensure complete removal of unused solvent and starting materials. As a final step, the suspension in hexane was also centrifuged to concentrate a small amount of dark green solid material, which could then be removed to obtain a colourless, transparent nanocrystal suspension.

Characterisation. Powder X-ray diffraction (XRD) patterns were collected from material drop cast onto a silicon zero background holder using a PANalytical X'Pert Pro diffractometer using Cu Kα radiation (λ = 1.5419 Å). Scanning transmission electron microscopy (STEM) samples were prepared by drop casting the nanoparticle dispersion in hexane directly onto a gold grid coated with an amorphous holey carbon film. STEM images and Energy dispersive X-ray (EDX) spectroscopy elemental maps were collected using a Thermo Fisher Titan STEM (G2 80-200) equipped with a Cs probe corrector (CEOS), high-angle annual dark-field (HAADF) detector and ChemiSTEM Super-X EDX detector, operating at 200 kV.

Optical Measurements. Optical absorption spectra were recorded using a PerkinElmer Lambda 1050 UV-visible spectrometer between 200-600 nm, and photoluminescence (PL) spectra were recorded on a Horiba Jobin Yvon Fluorolog-3 model FL3-22iHR spectrofluorometer with a monochromated Xe lamp. All measurements were recorded on nanocrystals suspended in hexane.

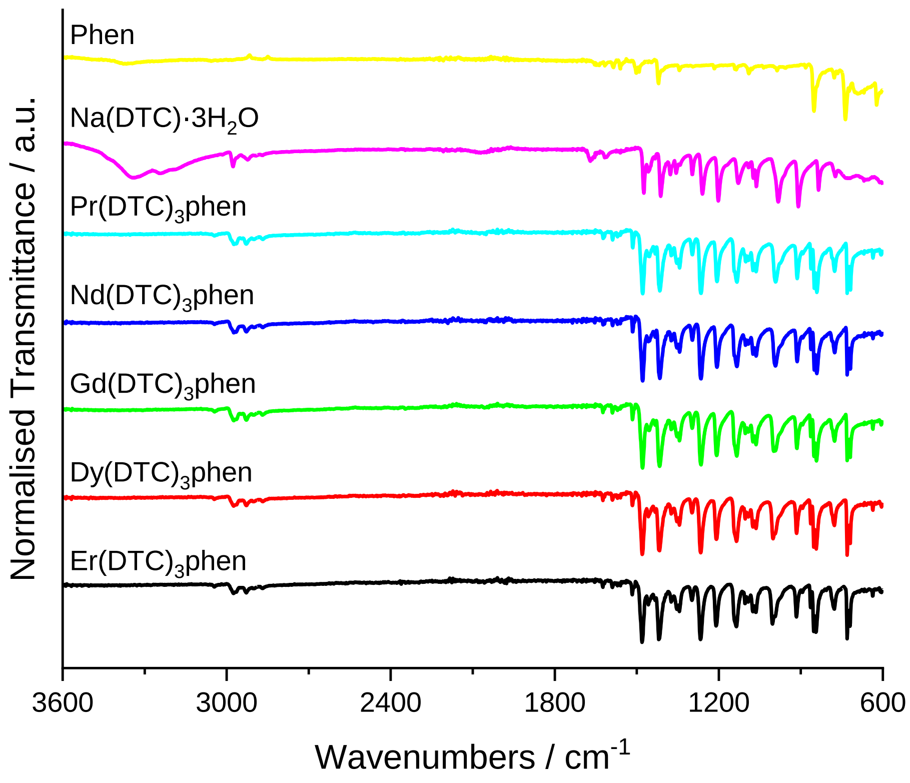

Figure S1: Fourier transform infrared (FTIR) spectra of the molecular precursors together with the spectra of the sodium dithiocarbamate salt and 1,10-phenanothroline reagents used to aid in assignment.

Calculation of the molar configurational entropy. The equation used to calculate the molar configurational entropy for a system where the entropic contribution comes from one sub-lattice is as follows:

$$S_{\mathrm{m}} = -R\frac{N^{\mathrm{Ln}}}{N}\sum \frac{N^{\mathrm{X}}}{N^{\mathrm{Ln}}}\ln\frac{N^{\mathrm{X}}}{N^{\mathrm{Ln}}}$$

where N is the total number of atoms in the system, N^{Ln} is the total number of lanthanide atoms, N^{X} is the number of atoms of lanthanide X and R is the ideal gas constant. This model assumes a perfectly random distribution of atoms throughout the material.

Williamson-Hall analysis. Williamson-Hall analysis allows for the deconvolution of strain- and size-related broadening to diffraction peaks in XRD measurements based on how they vary with θ. The peaks observed in the powder diffraction pattern are fitted to the formula:

$$\beta \cos\theta = 4\,\varepsilon \sin\theta + \frac{K\lambda}{D}$$

where β is the integral breadth of a diffraction peak, θ is the Bragg angle in radians, ε is the crystalline strain, K is the Scherrer constant, λ is the diffraction wavelength used, and D is the average crystallite size. The Scherrer constant was taken to be 0.89 in this work. A plot of $\beta\ \cos\theta$ vs. $\sin\theta$ allows the strain and crystallite size to be determined from the slope and intercept respectively.

We carried out this analysis using HighScore by first taking into account the instrumental broadening, then fitting peak functions, including a background, to our experimental data. Some peaks were discarded due to significant overlap or due to particularly poor fitting in the program.

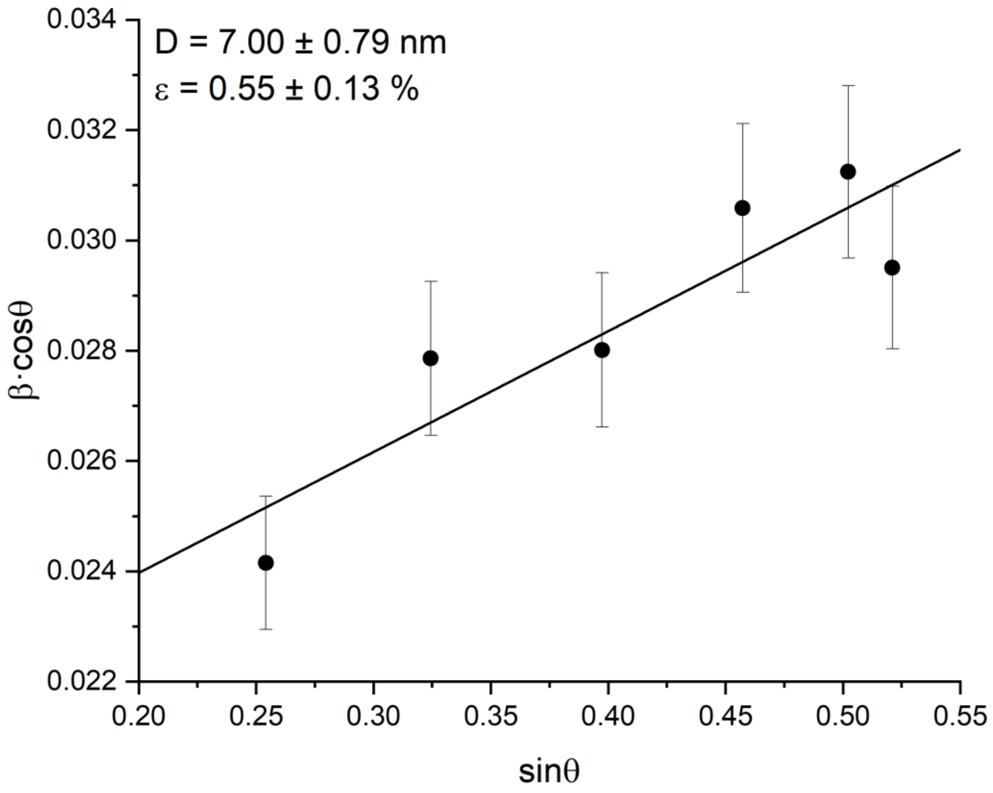

Figure S2. Williamson-Hall analysis of our XRD measurements showing the linear fit and calculated values of the average crystallite size D and strain ε. Error bars represent a nominal error of ±10%.

Further high-resolution STEM-EDX and STEM-EDX measurements.

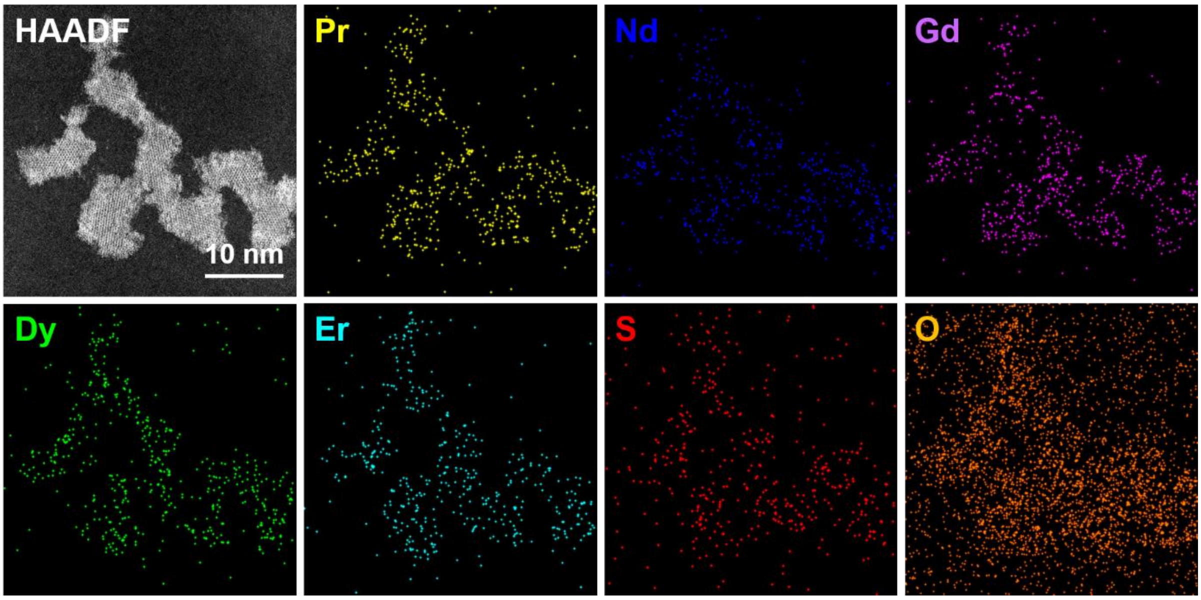

Figure S3: Higher-resolution STEM-EDX data collected on a small number of particles, confirming the presence of each element inside each particle. As the electron beam fluence (4.7×10^6 e/Å^2) was increased around 10 times compared to the Figure 3, the HAADF image shows clear electron beam damage to the sample during the time taken to collect these maps.

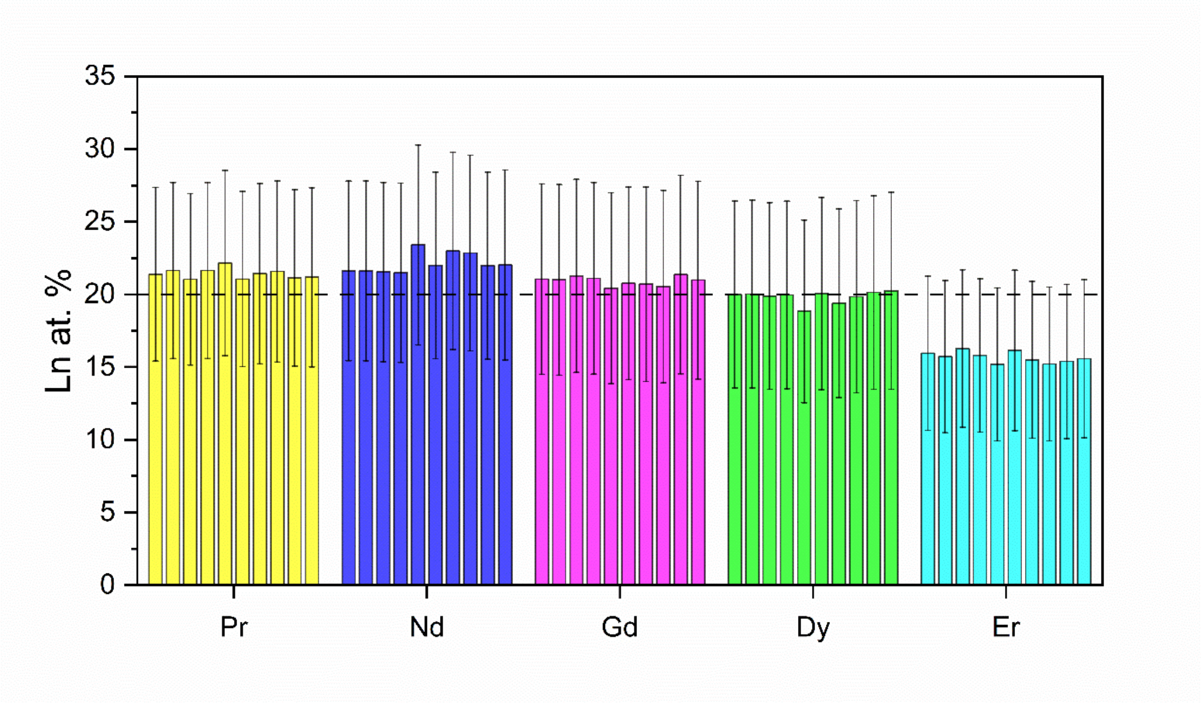

Figure S4: Lanthanide compositions measured using STEM-EDX on different areas of a nanoparticle sample. The instrumental errors are shown as error bars. The average X-ray counts were around 10^5 to 10^6 when measuring each area.

Photoluminescence lifetime measurement and characterization. A home-built time correlated single photon counting (TCSPC) system was used to investigate the photoluminescence (PL) decay transients. A mode-locked Ti:sapphire laser (Mai-Tai HP, Spectra Physics) is used to produce 100 fs pulses at a repetition rate of 80 MHz and 700 nm wavelength. The repetition rate is reduced to 4 MHz by an acousto-optic pulse picker (APE pulseSelect) and the initial wavelength halved to 350 nm *via* second harmonic generation (APE HarmoniXX). These pulses were used to excite the samples with an average power of 80 µW. The PL emission of the samples was collected and focused into a monochromator (Spex 1870c) and detected at the PL peak (410 nm) by a multi-channel plate (Hamamatsu R3809U-50). The time correlation of the detected photons was performed using a PC TCSPC electronics card (Edinburgh Instruments TCC900). Fitting was performed using a convolution of the system instrument response function (IRF) of approximately 50 ps. A PL trace of the solvent, hexane, was also measured, and found to be a significantly weaker signal with a lifetime shorter than the system IRF. This signal contribution was subtracted from the sample trace before fitting.

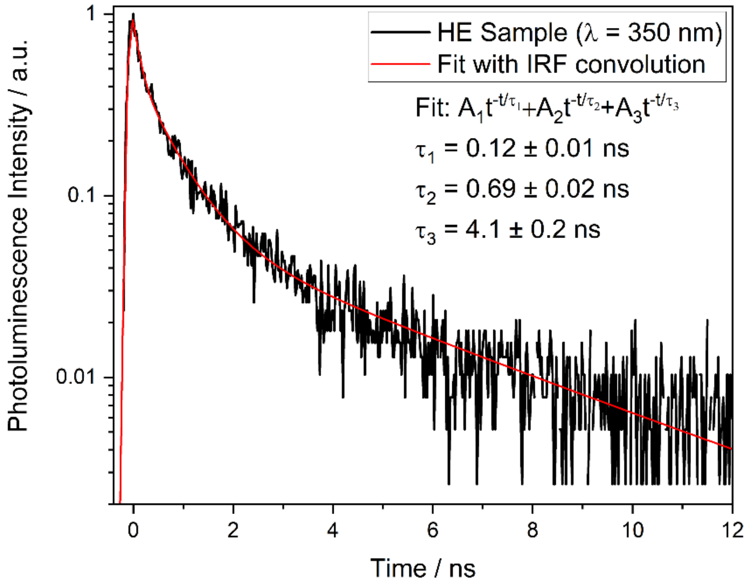

Figure S5: Photoluminescence decay trace (black line) of the nanoparticle suspension following excitation at 350 nm. After subtraction of the solvent contribution to the signal, a tri-exponential fit to the decay (red line) with a convolution to the system instrument response function (IRF) was generated. The lifetimes of the three components (designated τ_1, τ_2, and τ_3) are given in the figure, with relative amplitudes 0.12, 0.33, and 0.29 respectively.

Computational modelling. Computational modelling was carried out using pseudopotential plane-wave density functional theory (DFT) as implemented in the Vienna *Ab initio* Simulation Package (VASP) code.[1]

Initial models of the five bulk Ln_2SO_2 endpoints, *viz.* Pr_2SO_2, Nd_2SO_2, Gd_2SO_2, Dy_2SO_2 and Er_2SO_2, were prepared based on the Gd_2SO_2 structure from the Materials Project database[2] (**mp-4805**) and fully optimized using the PBEsol exchange-correlation functional with the DFT + U correction.[3, 4]

The band structures and dielectric constants (relative permittivities) ε_r were computed and used to estimate exciton Bohr radii a_b^* according to:

$$a_b^* = \varepsilon_r \left(\frac{m_e}{\mu}\right) a_0$$

where m_e is the electron rest mass, μ is the reduced mass computed from the electron and hole effective masses of the valence and conduction bands involved in the transition, and a_0 is the Bohr radius. We analyzed both the lowest-energy indirect transitions from K-A and the lowest-energy direct transitions at Γ. In both cases, due to the anisotropy of the crystals, we considered the effective masses along multiple k-space directions and computed the maximum and minimum reduced masses to obtain upper and lower bounds for the exciton radii.

To model the effect of volume strain on the electronic structure, a series of constant-volume optimizations were performed at expansions and compressions of ± 10 % of the optimised equilibrium volume in steps if 0.5 %. Accurate bandgaps were then calculated for a subset of the optimised structures, *viz.* ± 5 % of the equilibrium volume in steps of 1 %, using the HSE 06 hybrid functional.[5]

To model the effect of confinement and surface termination along the *c* direction, a series of slab models of the <001> surface were prepared consisting of 1-5 unit cells terminated with under-coordinated Ln atoms, with an initial vacuum gap of 15 Å between periodic images. These were then modified with one of two surface terminations, *viz.* formate (HCO_2^-) or acetate ($CH_3CO_2^-$). In each case the modification was applied to the "top" surface and replicated to the "bottom" surface using the inversion symmetry operation in order to avoid introducing a net dipole moment. The slab models were optimized as for the bulk cells using PBEsol + U but at fixed volume, i.e. allowing only the atomic coordinates to relax. For the larger slab models it was not feasible to calculate bandgaps using HSE 06, so we instead used the r^{2}SCAN meta-GGA functional.[6] Tests indicated that r^{2}SCAN underestimates the direct bandgaps of the Ln_2SO_2 endpoints by 14.14 $\pm$ 0.11 % and the bandgaps of several of the single-layer terminated surface slab models by 15.36 $\pm$ 5.31 %. This is a considerable improvement on PBEsol, which underestimates the gaps of the bulk and slab models by 27.08 $\pm$ 0.84 and 27.75 $\pm$ 2.4 %, respectively.

The ion cores were modelled with projector augmented-wave (PAW) pseudopotentials.[7, 8] For H, C, S and O we used the standard potentials including the outermost s and p electrons in the valence region. For the five Ln ions, we used the "Ln_3" potentials that place the unpaired electrons in the +3 oxidation state in the pseudopotential core. In the PBEsol + U calculations, a Hubbard U correction of 5 eV was applied to the Ln 4f states using the method in Ref. 4. A plane-wave basis set with a kinetic-energy cutoff of 750 eV was used to model the valence wavefunctions together with Γ-centered Monkhorst-Pack k-point meshes with $7 \times 7 \times 3$ (bulk)

and 7 × 7 × 1 subdivisions (surface slab).[9] The electronic wavefunctions were optimized to a tolerance of 10^{-8} eV on the total energy, and geometry optimizations were performed to a tolerance of 10^{-2} eV Å^{-1} on the forces. The precision of the charge-density grids was chosen automatically to avoid aliasing errors (`PREC = Accurate` in VASP), the PAW projection was performed in reciprocal space (`LREAL = .FALSE.`), and non-spherical contributions to the gradient corrections inside the PAW spheres were accounted for (`LASPH = .TRUE.`). For the HSE 06 calculations, reduced-precision grids were used to calculate the exact-exchange energy to improve performance (`PRECFOCK = Fast`).

The band structures of the equilibrium structures were calculated by interpolating between the eigenvalues obtained on a uniform dense *k*-point sampling mesh with 14 × 14 × 6 subdivisions using the AMSET code.[10] The high-frequency and ionic components of the dielectric constants were obtained using the density-functional perturbation theory and finite-differences routines in VASP[11] using the same dense *k*-point sampling as for the band structures. We note that while one would typically expect GGA calculations to underestimate bandgaps, they tend to yield reasonable band curvatures, and for calculating the effective masses required to compute the exciton radii the ability to use a denser *k*-point sampling mesh makes GGA calculations more suitable than hybrid calculations.

These calculated exciton radii are comparable to the thickness of the nanoparticles and have the same order of magnitude as the lateral plate size. These calculations do not take into account the effect of the alloying on the band gap. In previous work on bulk materials of the same composition it was found that the band gap of the alloyed material was significantly lower than the band gap of the mono-metal systems. It seems reasonable to suggest, then, that the alloying of the metals together could modulate the band structure and effective masses, and could affect the dielectric constant, increasing exciton radius towards the lateral size.

Table S1: The result of the exciton Bohr radius calculations. The ranges given are minimum/maximum bounds, calculated considering different combinations of valence/conduction band states.

Material	Direct / nm	Indirect / nm
Pr_2SO_2	1.26-2.82	1.35-2.33
Nd_2SO_2	1.32-2.94	1.35-2.31
Gd_2SO_2	1.66-3.82	1.34-2.24
Dy_2SO_2	1.84-4.11	1.34-2.20
Er_2SO_2	2.04-4.45	1.34-2.14

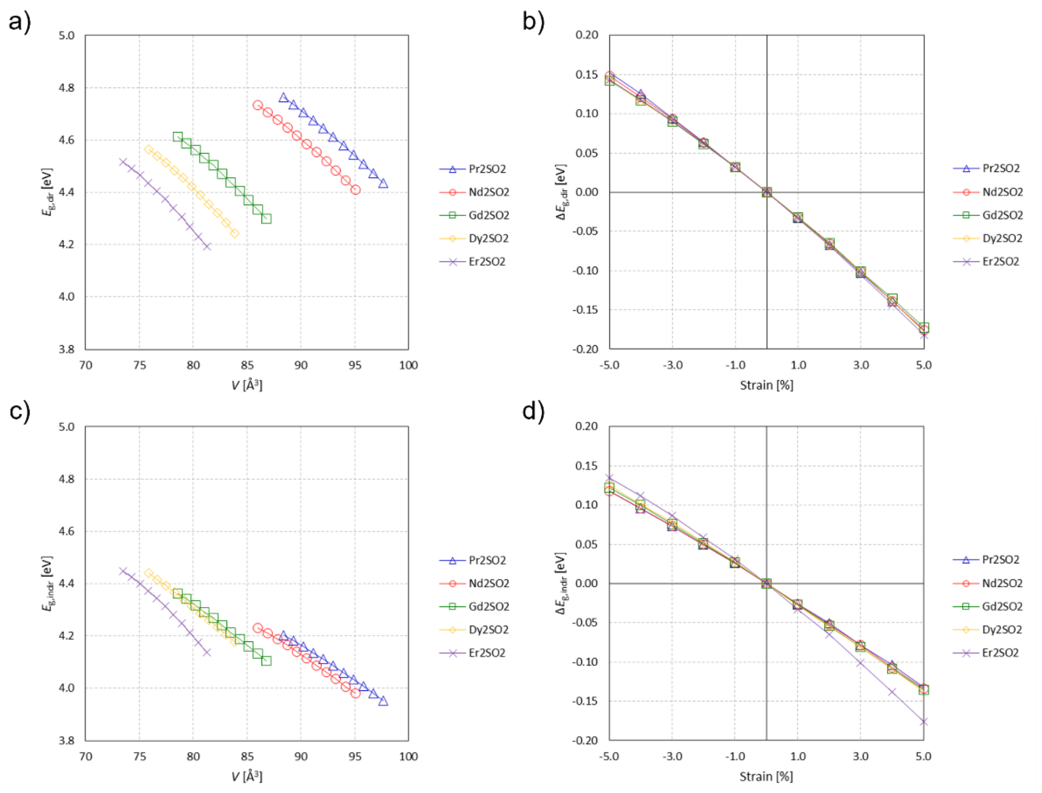

Figure S6: Variation of the direct (a) and indirect (c) band gaps of the five Ln_2SO_2 endpoints, *viz.* Pr_2SO_2, Nd_2SO_2, Gd_2SO_2, Dy_2SO_2 and Er_2SO_2, with unit cell volume, and corresponding change in the direct (b) and indirect (d) bandgaps as a function of volume strain.

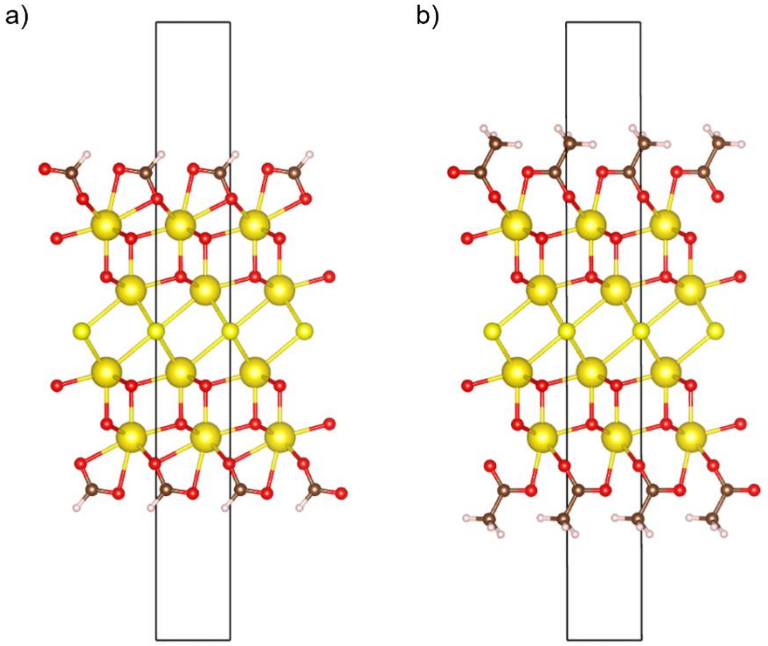

Figure S7: Representative images of optimized bilayer slab models capped with (a) formate and (b) acetate.

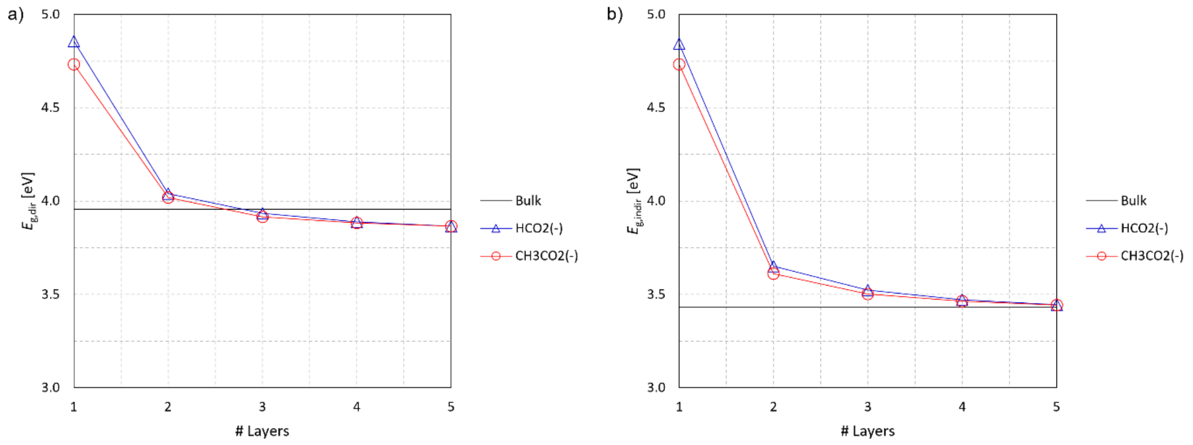

Figure S8: Variation of the direct (a) and indirect (b) bandgaps of Pr_2SO_2 slab models capped with formate ($[HCO_2]^-$) and acetate ($[CH_3CO_2]^-$) with the number of layers. On each plot the solid black line shows the bandgap calculated for bulk Pr_2SO_2 for comparison.

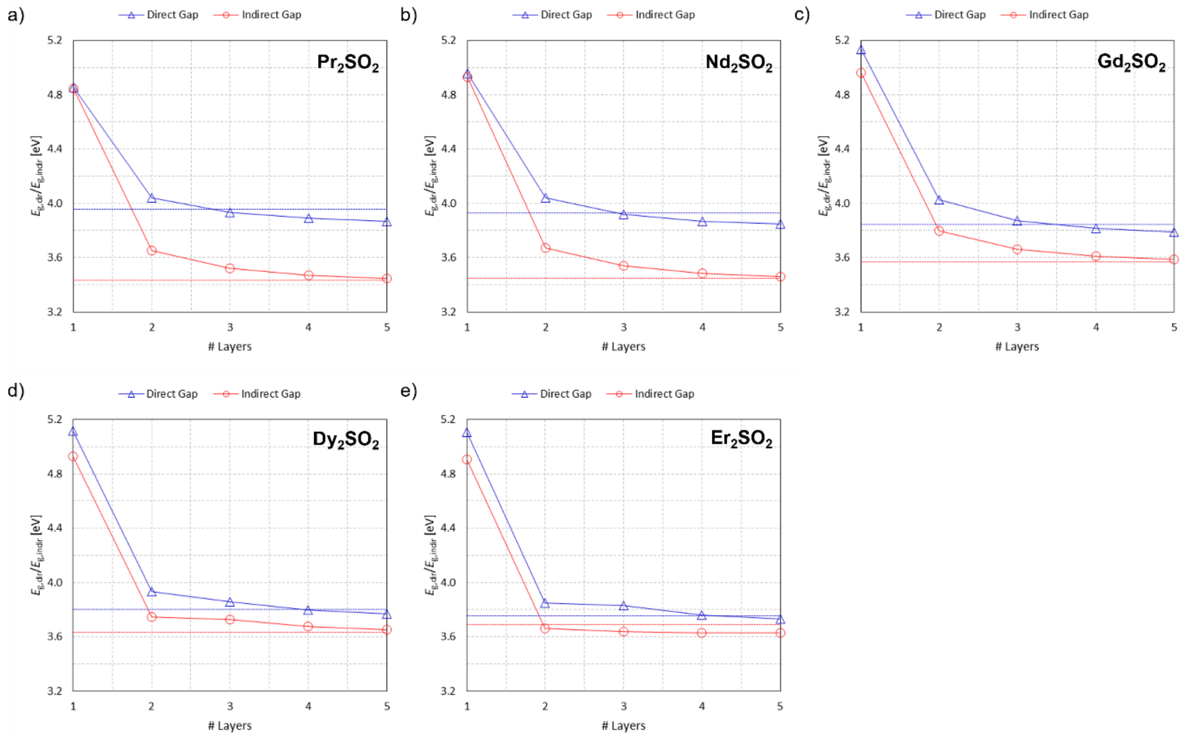

Figure S9: Variation of the direct and indirect bandgaps of slab models of the five Ln_2SO_2 endpoints, *viz.* Pr_2SO_2 (a), Nd_2SO_2 (b), Gd_2SO_2 (c), Dy_2SO_2 (d) and Er_2SO_2 (e), capped with formate with the number of layers. The dashes colored lines show the bandgaps of the corresponding bulk materials for comparison.

1. Kresse, G.; Hafner, J., Ab initio molecular dynamics for liquid metals. *Phys. Rev. B* **1993,** *47* (1), 558-561.
2. Jain, A.; Ong, S. P.; Hautier, G.; Chen, W.; Richards, W. D.; Dacek, S.; Cholia, S.; Gunter, D.; Skinner, D.; Ceder, G.; Persson, K. A., Commentary: The Materials Project: A materials genome approach to accelerating materials innovation. *APL Mater.* **2013,** *1* (1), 011002.
3. Perdew, J. P.; Ruzsinszky, A.; Csonka, G. I.; Vydrov, O. A.; Scuseria, G. E.; Constantin, L. A.; Zhou, X.; Burke, K., Restoring the Density-Gradient Expansion for Exchange in Solids and Surfaces. *Phys. Rev. Lett.* **2008,** *100* (13), 136406.
4. Dudarev, S. L.; Botton, G. A.; Savrasov, S. Y.; Humphreys, C. J.; Sutton, A. P., Electron-energy-loss spectra and the structural stability of nickel oxide: An LSDA+U study. *Phys. Rev. B* **1998,** *57* (3), 1505-1509.
5. Krukau, A. V.; Vydrov, O. A.; Izmaylov, A. F.; Scuseria, G. E., Influence of the exchange screening parameter on the performance of screened hybrid functionals. *J. Chem. Phys.* **2006,** *125* (22), 224106.
6. Furness, J. W.; Kaplan, A. D.; Ning, J.; Perdew, J. P.; Sun, J., Accurate and Numerically Efficient r2SCAN Meta-Generalized Gradient Approximation. *J. Phys. Chem. Lett.* **2020,** *11* (19), 8208-8215.
7. Kresse, G.; Joubert, D., From ultrasoft pseudopotentials to the projector augmented-wave method. *Phys. Rev. B* **1999,** *59* (3), 1758-1775.
8. Blöchl, P. E., Projector augmented-wave method. *Phys. Rev. B* **1994,** *50* (24), 17953-17979.
9. Monkhorst, H. J.; Pack, J. D., Special points for Brillouin-zone integrations. *Phys. Rev. B* **1976,** *13* (12), 5188-5192.
10. Ganose, A. M.; Park, J.; Faghaninia, A.; Woods-Robinson, R.; Persson, K. A.; Jain, A., Efficient calculation of carrier scattering rates from first principles. *Nat. Commun.* **2021,** *12* (1), 2222.
11. Gajdoš, M.; Hummer, K.; Kresse, G.; Furthmüller, J.; Bechstedt, F., Linear optical properties in the projector-augmented wave methodology. *Phys. Rev. B* **2006,** *73* (4), 045112.
